# *SP1* and *KROX20* Regulate the Proliferation of Dermal Papilla Cells and Target the *CUX1* Gene

**DOI:** 10.3390/ani14030429

**Published:** 2024-01-29

**Authors:** Xiaoyang Lv, Mingliang He, Hui Zhou, Shanhe Wang, Xiukai Cao, Zehu Yuan, Tesfaye Getachew, Yutao Li, Wei Sun

**Affiliations:** 1 Joint International Research Laboratory of Agriculture and Agri-Product Safety of Ministry of Education of China, Yangzhou University, Yangzhou 225009, China; dx120170085@yzu.edu.cn (X.L.); yuanzehu1988@163.com (Z.Y.); 2 International Joint Research Laboratory in Universities of Jiangsu Province of China for Domestic Animal Germplasm Resources and Genetic Improvement, Yangzhou University, Yangzhou 225009, China; 3 College of Animal Science and Technology, Yangzhou University, Yangzhou 225009, China; 4 International Centre for Agricultural Research in the Dry Areas, Addis Ababa 999047, Ethiopia; 5 CSIRO Agriculture and Food, 306 Carmody Rd, St Lucia, QLD 4067, Australia

**Keywords:** *CUX1*, transcriptional regulatory, *SP1*, *KROX20*, DPCs proliferation

## Abstract

**Simple Summary:**

Dermal papilla cells (DPCs) are an important cell in the hair follicle that can maintain nutrition and induce hair follicle formation. A previous study demonstrated that CUT-like homeobox 1 (*CUX1*) could promote the proliferation of ovine DPCs, but the upstream transcriptional regulatory mechanisms of the *CUX1* gene remain largely unknown. In the present study, we aim to investigate the upstream transcriptional regulators of *CUX1* to enhance our comprehension of the mechanism of the *CUX1* gene in ovine DPCs. Our findings demonstrate that *SP1* and *KROX20* are two upstream transcriptional regulators of *CUX1* and play a critical role in regulating the proliferation of ovine DPCs.

**Abstract:**

Previous studies have demonstrated that *CUX1* could contribute to the proliferation of DPCs in vitro, but the upstream transcriptional regulatory mechanisms of *CUX1* remain largely unknown. This study aimed to investigate the upstream transcriptional regulators of *CUX1* to enhance our comprehension of the mechanism of action of the *CUX1* gene in ovine DPCs. Initially, the JASPAR (2024) software was used to predict the upstream target transcription factors for the *CUX1* gene. Subsequently, through RT-qPCR and a double luciferase reporter assay, the interaction between *SP1*, *KROX20*, and *CUX1* was established, respectively. The results indicated that *SP1* and *KROX20* were two highly reliable upstream transcription regulators for the *CUX1* gene. Additionally, we found that *SP1* promoted the proliferation of DPCs by overexpressing *SP1* in DPCs, and *KROX20* inhibited the proliferation of DPCs by overexpressing *KROX20* in DPCs. These findings are also consistent with the transcriptional regulation of *CUX1* by *SP1* and *KROX20*, respectively. This study suggests that the effect of DPC proliferation in vitro by *CUX1* may regulated by the transcription factors *SP1* and *KROX20*.

## 1. Introduction

The wool trait is an important economic trait, and the wool industry derived from it plays an important role in the national economy. Hu sheep, a distinct breed indigenous to China, has a breeding history of nearly 900 years in China, and it has many excellent characteristics, such as good meat performance, multi-purpose fur, high milk quality, and strong adaptability to the environment. Additionally, Hu sheep are renowned for their ability to produce patterned wool, which mainly includes two wool phenotypes, curly wool phenotypes and straight wool phenotypes. Therefore, it is highly significant to study the traits of wool produced by Hu sheep. Wool grows from the hair follicle (HF), which serves as the foundation for hair growth and regulation [1]. The structure of hair follicles is columnar and runs through the epidermis and dermis. Studies have found that the interaction between the two cell types influences the growth and development of hair follicles [2,3]. Specifically, the dermis is composed of the dermal papilla (DP) and dermal sheath (DS) [4], while the epidermis consists of the matrix (Mx), hair shaft (HS), inner root sheath (IRS), and outer root sheath (ORS) [5,6]. At the base of the hair follicle, there is an enlarged region called the hair bulb (HB), which governs the circulation and growth of the hair follicle [7]. The central concave part of the hair bulb is known as the hair papilla, which is responsible for regulating the number of hair progenitor cells and thus influencing hair growth [8]. Dermal papilla cells (DPCs) possess unique clumping growth characteristics, and they play a vital role in inducing hair follicle formation as well as serving as a source of nutrients for the hair follicles [9].

CUT-like homeobox 1 (*CUX1*) is a member of the DNA-binding protein homologous domain family, which is also known as CASP, CUTL1, CDP, or CUX [10]. Numerous studies have demonstrated the crucial role of the *CUX1* gene in organ development and the maintenance of internal environmental stability. Inactivation of the *CUX1* gene in mice has been found to result in various abnormal phenotypes, including growth retardation, delayed differentiation of lung epithelial cells, morphological changes in hair follicles and curly whiskers, male sterility, and defects in T and B cells [11,12,13]. As an important transcription factor, *CUX1* can stably bind to DNA and inhibit or promote the transcription of some genes [14,15]. Ellis et al. conducted targeted mutations in mice to disrupt CDP’s ability to inhibit gene transcription and revealed that mice with *CUTL1* mutations experienced delayed differentiation of lung epithelial cells and suffered from premature death shortly after birth, whereas surviving mice exhibited stunted growth and abnormal hair development [11]. Our previous study discovered that the *CUX1* gene can enhance the proliferation of Hu sheep DPCs in vitro. Nonetheless, limited comprehension exists regarding the upstream transcriptional regulatory mechanisms governing the *CUX1* gene.

Transcription factors regulate gene expression, which in turn affects the formation of traits. Thus, it is important to investigate the regulatory effects of transcription factors on key genes to analyze trait formation. Numerous studies have demonstrated the involvement of transcription factors in the regulation of hair follicle growth and development. By analyzing the expression of *Sox10* in hair morphogenesis, the postnatal hair cycle, and the hair follicle cycle induced by hair removal in mice, Jing et al. speculated that *Sox10* may play a role in early hair follicle morphogenesis and the postnatal hair follicle cycle [16]. Furthermore, *NF-κB*, a transcription factor, has been found to participate in the control of the mouse hair cycle and exhibits varied roles in different types of hair follicles. It controls the expression of transcription factors *Sox9* and *Lhx2*, which are responsible for maintaining and activating hair follicle stem cells [17,18,19,20]. For sheep wool traits, transcription factor *MZF1* negatively regulates the promoter region of the *KRT71* gene, leading to the disappearance of the hair curl trait in Tan sheep [21]. These studies demonstrate that transcription factors not only have a role in self-regulating hair follicle development but also influence the regulation of hair follicle development by target genes.

In this study, we aimed to investigate the upstream transcriptional regulatory mechanism of the *CUX1* gene to understand the regulation of *CUX1* gene upstream transcription factors on *CUX1* expression and DPCs proliferation, to enhance our comprehension of the mechanism of the *CUX1* gene in the regulation of DPC proliferation.

## 2. Materials and Methods

### 2.1. Ethics Statement

According to the “Jiangsu Province Laboratory Animal Management Measures”, this experiment was designed and approved by the Animal Ethics Committee of Yangzhou University (Approval number: No. 202103279).

### 2.2. Animals, Cell Isolation, Cell Culture, and Cell Transfection

The DPCs used in this experiment were isolated from the wool follicles of a healthy 3-day-old lamb of Hu sheep, provided by the Suzhou Sheep Farm (Suzhou, China). The isolation protocol for DPCs was adopted from our previous study [22]. The culture medium used for the DPCs was DMEM-F12 (Sigma-Aldrich, St. Louis, MO, USA) supplemented with 10% fetal bovine serum (Gibco, Grand Island, NY, USA) and 1% penicillin–streptomycin–amphotericin (Solar-bio, Beijing, China). The cells were cultured under the condition of 5% CO_2_ and 37 °C. For cell transfection, we used the ietPRIME Transfection Reagent (Polyplus, Illkirch, France) following the instructions provided by the manufacturer.

### 2.3. Total RNA Extraction, cDNA Synthesis, and qRT-PCR

We extracted total RNA from DPCs using the TRNzol Universal reagent (TIANGEN, Beijing, China); RNA concentration and purity were determined using a spectrophotometer (Thermo, Waltham, MA, USA). We synthesized the cDNA of target genes using a reverse transcription kit (TIANGEN, Beijing, China) and detected the expression of target genes using 2 × TSINGKE Master qPCR Mix (Tsingke, Nanjing, China). The housekeeping gene used was Sheep GAPDH and gene expression was calculated using the 2^−ΔΔCT^ method [23].

### 2.4. Primers for qRT-PCR

In this experiment, the primers for quantitative detection were designed using the BLAST tool available on the NCBI online website and synthesized by Tsingke (Nanjing, China). Information on all primers is provided in Table 1.

### 2.5. Construction of SP1 and KROX20 Overexpression Vector and CUX1 Promoter Reporter Vector

The full-length sequence of the *SP1* and *KROX20* coding sequence (CDS) region was amplified using cDNA extracted from Hu sheep skin tissue as a template, and the amplified fragment was constructed into the pcDNA 3.1 vector using homologous recombination technology, followed by confirmation of the vector via sequencing. These restriction sites were EcoRI and HindIII. The successfully constructed *SP1* and *KROX20* overexpression vectors were named pcDNA3.1-SP1 and pcDNA3.1-KROX20, respectively.

The sequence of the *CUX1* core promoter region was amplified using DNA extracted from Hu sheep skin tissue as a template, and the amplified fragment was constructed into the pGL3-basic vector using homologous recombination technology, followed by confirmation of the vector via sequencing. These restriction sites were XhoI and HindIII. The successfully constructed *CUX1* promoter reporter vector was named pGL3-CUX1-1601. All primers used for vector conduction were synthesized by Tsingke (Nanjing, China). Information on all primers is provided in Table 2.

### 2.6. Immunofluorescence

The DPCs were cultured in a 24-well plate. First, the DPCs were washed using 1X PBS (Solarbio, Beijing, China) and were fixed with a 4% paraformaldehyde solution (Solarbio, Beijing, China). Then, we used 0.5% Triton X-100 (Solarbio, Beijing, China) to permeate the DPCs and 5% BSA (Solarbio, Beijing, China) to incubate the DPCs. Finally, we used the primary antibody and the secondary antibody to incubate the DPCs, respectively. DAPI (Beyotime, Shanghai, China) was used to stain the nuclei of the DPCs. An inverted fluorescence microscope (Nikon, Tokyo, Japan) was utilized to capture images. The primary antibodies were an ACTA1 rabbit polyclonal antibody (Sangon Biotech, Shanghai, China, 1:400, D121592), a PDGFRA rabbit monoclonal antibody (Abcam, Cambridge, UK, 1:400, ab203491), and an SOX18 rabbit polyclonal antibody (Bioss, Beijing, China, 1:400, bs-17135R). The secondary antibody was Goat Anti-Rabbit IgG H&L (Alexa Fluor^®^ 555) (Abcam, Cambridge, UK, 1:400, Ab150078).

### 2.7. Dual-Luciferase Assay

The DPCs were cultured in a 24-well plate until they reached 50% cell density. We then transfected them using the ietPRIME Transfection Reagent (Polyplus, Illkirch, France). All dosages and procedures for transfection were strictly followed based on the manufacturer’s instructions. After 24 h of transfection, we processed the DPCs using a dual-luciferase detection kit (Vazyme, Nanjing, China) following the manufacturer’s instructions. The Firefly luciferase activity and the Renilla luciferase activity were measured using a Microplate Reader (EnSpire, PerkinElmer, Waltham, MA, USA).

### 2.8. CCK-8 Assay

The DPCs were cultured in a 96-well plate until they reached 30% cell density. We then transfected them using the ietPRIME Transfection Reagent (Polyplus, Illkirch, France). All dosages and procedures for transfection were strictly followed based on the manufacturer’s instructions. Cell viability was assessed at 0 h (12 h after transfection), 24 h, 48 h, and 72 h using the CCK-8 Kit (Vazyme, Nanjing, China), following the manufacturer’s instructions. The absorbance of the DPCs at 450 nm was measured using a Microplate Reader (EnSpire, Perkin Elmer, Waltham, MA, USA).

### 2.9. EdU Assay

The DPCs were cultured in a 24-well plate until they reached 50% cell density. We then transfected them using the ietPRIME Transfection Reagent (Polyplus, Illkirch, France). All dosages and procedures for transfection were strictly followed based on the manufacturer’s instructions. After 24 h of transfection, we processed DPCs using an EdU Apollo In Vitro Imaging Kit (RiboBio, Guangzhou, China) following the manufacturer’s instructions. Images of stained cells were captured using a fluorescence-inverted microscope (Nikon, Tokyo, Japan). The Image Pro Plus 6.0 software (Media Cybernetics, Rockville, MD, USA) was used for image analysis.

### 2.10. Statistical Analysis

The relative expression levels of related genes were calculated using the 2^−ΔΔCT^ method, and a T-test was used for analysis. The activity values of the CUX1 core promoter region were analyzed using the ANOVA method. The GraphPad Prism 8 software was used for data analysis and charting. The results are expressed as means ± SEM (standard error of the mean). *p* < 0.05 indicates a significant difference (represented by “*”), *p* < 0.01 indicates a very significant difference (represented by “**”), and *p* < 0.001 indicates an extremely significant difference (represented by “***”). Each experiment in this study involved three biological replicates.

## 3. Results

### 3.1. Identification of Marker Genes in Hu Sheep DPCs

**Figure 1:**
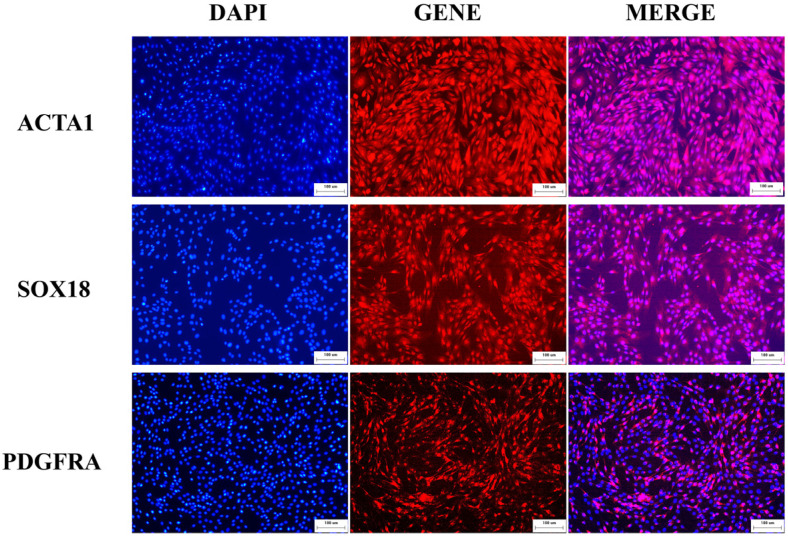
Identification of marker genes in Hu sheep DPCs; the scale is 100 µm.

To identify whether the isolated cells were Hu sheep DPCs, we used immunofluorescence staining to identify the purity of the DPCs. According to previous studies, we selected *α-SMA*(*ACTA1*) [24], *SOX18* [25], and *PDGFRA* [25] as three marker genes of Hu sheep DPCs. The results of the immunofluorescence staining indicated that the purity of the isolated cells was high and could be used for subsequent experiments (Figure 1).

### 3.2. Analysis of Transcription Factor Binding Sites on the CUX1 Gene

**Figure 2:**
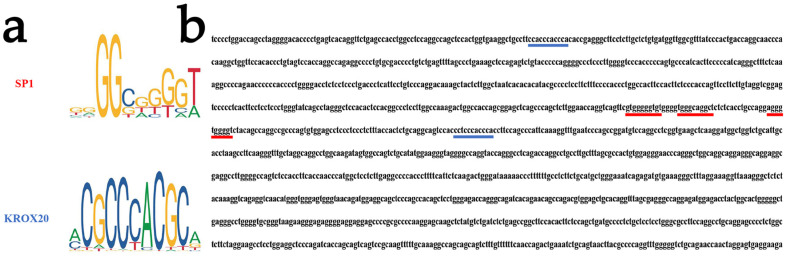
Bioinformatics analysis of transcription factor binding sites on the *CUX1* gene. (**a**) The binding motifs of transcription factors *SP1* and *KROX20* were searched on the website http://jaspar.genereg.net/ (accessed on 21 November 2023). (**b**) The core promoter region of the *CUX1* gene contains transcription factor binding sites for *SP1* and *KROX20*. Red and blue underlines represent transcription binding sites for the SP1 and KROX20, respectively.

To examine the specific effect of transcription factors on the *CUX1* gene, we performed a bioinformatics analysis using the JASPAR (2024) software. Our research found the presence of binding sites for the *SP1* and *KROX20* transcription factors in the promoter region of the *CUX1* gene (Figure 2a,b). Additionally, they were involved in regulating hair follicle growth and development [26,27,28].

### 3.3. SP1 and KROX20 Act as Two Transcription Regulatory Factors of the CUX1 Gene

**Figure 3:**
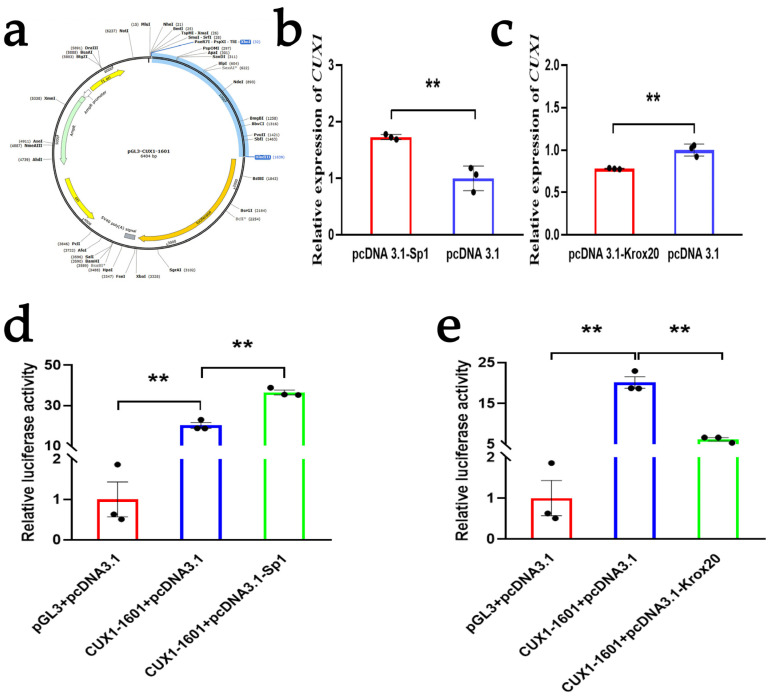
*SP1* and *KROX20* act as two transcription regulatory factors of the *CUX1* gene. (**a**) Sketch map of pGL3-CUX1-1601. *CUX1* cDNA sequence is inserted into the pGL3-basic vector by restriction enzyme cutting sites of XhoI and HindIII. (**b**,**c**) The mRNA expression of *CUX1* after DPCs were treated with an overexpression vector of *SP1* and *KROX20*, respectively. (**d**,**e**) The dual-luciferase assay for the activity of the *CUX1* core promoter region after co-transfecting the *CUX1* core promoter region reporter plasmids with pcDNA3.1-SP1 and pcDNA3.1-KROX20, respectively. The results are expressed as means ± SEM (standard error of the mean). *p* < 0.01 indicates a very significant difference (represented by “**”). Each experiment in this study involved three biological replicates.

To understand the regulation effect of *SP1* and *KROX20* on *CUX1*, we used qRT-PCR to detect changes in the *CUX1* expression levels after the overexpression of *SP1* and *KROX20* in DPCs, respectively. The results showed that the overexpression of *SP1* resulted in a significant increase in *CUX1* mRNA expression, while the overexpression of *KROX20* resulted in a significant decrease in *CUX1* mRNA expression (Figure 3b,c). In a previous study, we confirmed the core promoter region of the *CUX1* gene [29]. To further verify whether *SP1* and *KROX20* regulate the expression of *CUX1* by affecting the activity of the *CUX1* core promoter region, we first determined the luciferase reporter vector of the *CUX1* core promoter (Figure 3a). Then, we co-transfected the reporter plasmids (pGL3-CUX1-1601) with pcDNA3.1-SP1 and pcDNA3.1-KROX20, respectively. The results of the dual-luciferase assay demonstrated that the overexpression of *SP1* enhanced the activity of the *CUX1* core promoter region, while the overexpression of *KROX20* suppressed the activity of the *CUX1* core promoter region (Figure 3d,e). The results of the qRT-PCR are consistent with those of the dual-luciferase reporter system, further indicating that *SP1* and *KROX20* were two highly reliable upstream transcription regulators for the *CUX1* gene.

### 3.4. SP1 Enhances the Proliferation of DPCs

**Figure 4:**
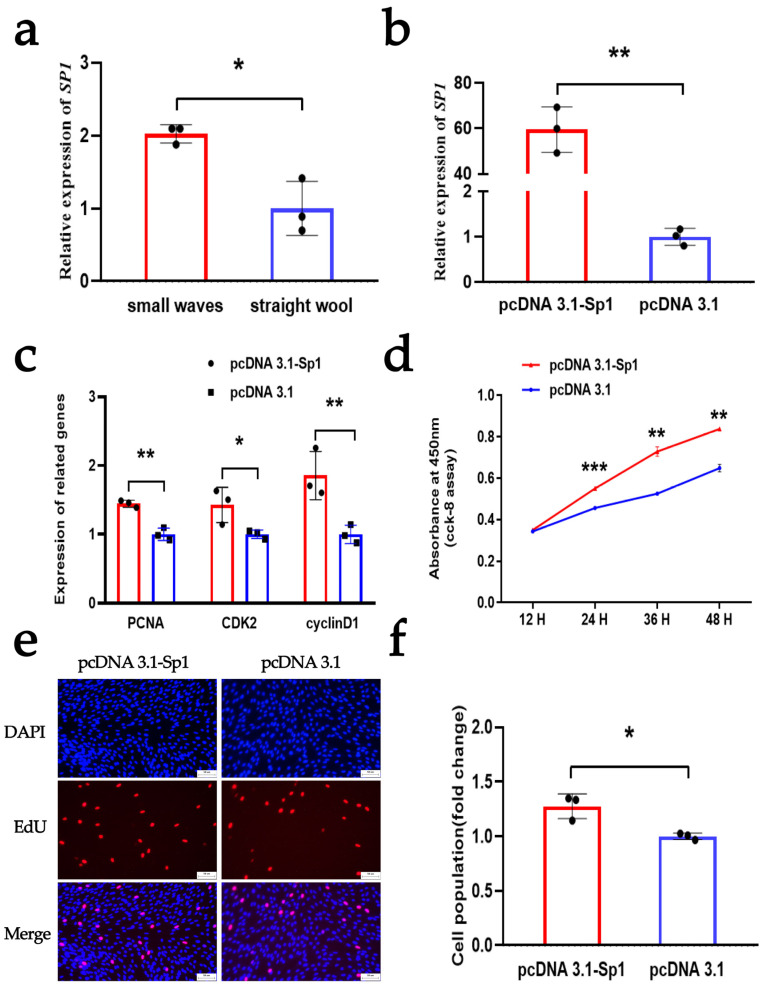
*SP1* promotes the proliferation of DPCs. (**a**) Expression levels of *Sp1* between small waves and straight wool. (**b**) The mRNA expression of *SP1* after DPCs were treated with an overexpression vector of *SP1*. (**c**) The mRNA expression of *PCNA*, *CDK2*, and *cyclinD1* after DPCs were treated with an overexpression vector of *SP1*. (**d**) CCK-8 assay after DPCs were treated with an overexpression vector of *SP1*. (**e**,**f**) EdU assay after DPCs were treated with an overexpression vector of *SP1*; the scale is 50 µm. The results are expressed as means ± SEM (standard error of the mean). *p* < 0.05 indicates a significant difference (represented by “*”), *p* < 0.01 indicates a very significant difference (represented by “**”), and *p* < 0.001 indicates an extremely significant difference (represented by “***”). Each experiment in this study involved three biological replicates.

To investigate the regulation of *SP1* on the proliferation of DPCs, we first detected the expression of *SP1* in DPCs isolated from the hair follicles of Hu sheep with different wool phenotypes. The qRT-PCR assay indicated that *SP1* has a high expression in DPCs isolated from the hair follicles of Hu sheep with small-wave wool phenotypes, which was consistent with the expression pattern observed for *CUX1* (Figure 4a). Subsequently, we successfully constructed an overexpression plasmid for *SP1* to study its function in Hu sheep DPCs. Following this, the plasmid was transfected into DPCs, and the mRNA expression of *SP1* was assessed. We observed a significant increase in the mRNA expression level of *SP1* in the qRT-PCR assay (Figure 4b). These results demonstrated the successful construction of the *SP1* overexpression vector, which could be used in subsequent experiments. To further elucidate the effect of *SP1*, we assessed its effect on the proliferation of DPCs using qRT-PCR, CCK-8, and EdU. The qRT-PCR assay showed that the overexpression of *SP1* could significantly increase the mRNA level of *PCNA*, *CDK2*, and *cyclinD1* (Figure 4c). The CCK8 and EdU assays showed that the overexpression of *SP1* could facilitate cell vitality and cell proliferation, respectively (Figure 4d–f). These findings suggest that the overexpression of *SP1* positively influences DPC proliferation. Furthermore, our results indicate that *SP1* and *CUX1* possess similar regulatory roles in the proliferation of DPCs.

### 3.5. KROX20 Suppresses the Proliferation of DPCs

**Figure 5:**
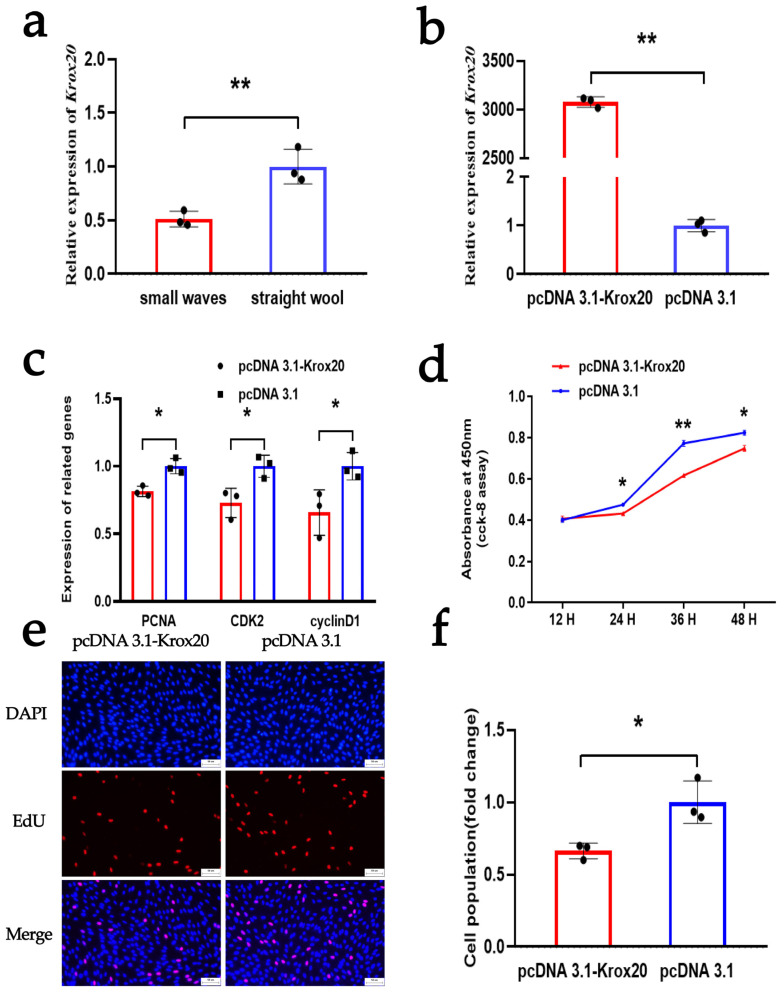
*KROX20* inhibits the proliferation of DPCs. (**a**) Expression levels of *KROX20* between small waves and straight wool. (**b**) The mRNA expression of *KROX20* after DPCs were treated with an overexpression vector of *KROX20*. (**c**) The mRNA expression of *PCNA*, *CDK2*, and *cyclinD1* after DPCs were treated with an overexpression vector of *KROX20*. (**d**) CCK-8 assay after DPCs were treated with an overexpression vector of *KROX20*. (**e**,**f**) EdU assay after DPCs were treated with an overexpression vector of *KROX20*; the scale is 50 µm. The results are expressed as means ± SEM (standard error of the mean). *p* < 0.05 indicates a significant difference (represented by “*”), and *p* < 0.01 indicates a very significant difference (represented by “**”). Each experiment in this study involved three biological replicates.

Similarly, we investigated the regulation of *KROX20* on the proliferation of DPCs. The qRT-PCR assay indicated that *KROX20* has a high expression in DPCs isolated from the hair follicles of Hu sheep with straight wool phenotypes, which was opposite to the expression pattern observed for *CUX1* (Figure 5a). Furthermore, we successfully constructed an overexpression plasmid for *KROX20* and transfected it into the DPCs. Subsequently, a qRT-PCR assay demonstrated a significant increase in the mRNA expression level of *KROX20* (Figure 5b). Following this, we assessed its effect on the proliferation of DPCs using qRT-PCR, CCK-8, and EdU. The qRT-PCR assay showed that the overexpression of *KROX20* could significantly decrease the mRNA level of *PCNA*, *CDK2*, and *cyclinD1* (Figure 5c). Moreover, the CCK8 and EdU assays showed that the overexpression of *KROX20* could inhibit cell vitality and cell proliferation, respectively (Figure 5d–f). These findings suggest that the overexpression of *KROX20* has a negative influence on the proliferation of DPCs. Furthermore, our results indicate that *KROX20* and *CUX1* possess opposite regulatory roles in the proliferation of DPCs.

## 4. Discussion

DPCs consist of a cluster of specialized fibroblasts; they play a critical role in hair formation, growth, and hair circulation [30]. Additionally, these cells possess dermal fibroblasts, but they also have different characteristics from fibroblasts, such as the agglutinative growth characteristic of DPCs. The agglutination property of DPCs is considered to play a key role in inducing hair follicle formation [31]. It was also reported that DPCs implanted under the skin alone can form complete hair follicles and produce hair [30]. Hair follicles are usually formed through the aggregation of dermal mesenchymal cells in embryonic skin, and the interaction between hair follicle epithelial cells and dermal mesenchymal cells plays an important role in the proliferation and differentiation of hair follicle cells and hair growth [32]. During the alternating cycle of hair follicle growth, DPCs can provide a variety of signaling regulatory factors for hair follicle growth and development, among which the most important ones are fibroblast growth factor 7 (*FGF7*), insulin growth factor 1 (*IGF1*) and protease inhibitor *Nexin-1*, all of which are secreted by DPCs. *FGF7* can induce keratinocyte proliferation, and *IGF1* can promote the proliferation of melanocytes, epithelium, and dermal cells. *Nexin-l* gene expression is directly related to the induction ability of DPCs and is an important pathway involved in the induction of hair follicle growth mediated by DPCs [33].

Studies have shown that the uneven distribution of dermal papilla at the bottom of the hairball leads to a difference in the growth rate of hair parent cells, which is an important factor in the regulation of hair types. Hair shafts are derived from keratinocytes in the epithelial cells of hair follicles, but the growth and differentiation of keratinocytes in hair follicles are guided by the hairy papilla embedded in the base of the hairball. DPCs are the regulatory center of hair follicle development, and the number and type of DPCs play a decisive role in hair follicle growth, wool type, and fleece [34,35]. Chi et al. found that four different hair types can be produced in a single hair follicle growth and development cycle in mice, including guard hair, cone hair, Duchene hair, and zigzag hair. Moreover, it has been observed that the difference in the shape of the dermal papilla and the number of DPCs in it is directly related to the type of hair, and with the reduction in the number of DPCs, the length and thickness of the hair produced are significantly reduced [35]. In addition, some researchers believe that the main reason for hair follicle curvature is the independent operation of multiple papillary centers (MPCs) formed in the dermal papilla, which is likely to cause asymmetric hair growth and curvature [5]. Curly hair originates from hairballs surrounded by many proliferating cells, due to the uneven distribution of their proliferating space [36].

In our previous study, we discovered that the *CUX1* gene could enhance the proliferation of DPCs. This finding suggests that the *CUX1* gene may play a role in the growth and development of hair follicles by influencing the proliferation of DPCs. However, the exact regulatory mechanism of the *CUX1* gene is unknown. To further understand the regulatory mechanism of the *CUX1* gene in the proliferation process of DPCs, we explored the upstream transcriptional regulatory mechanism of the *CUX1* gene in this study. In the process of molecular regulation, the regulation of transcription factors plays an important role in gene expression [37]. Therefore, we explored upstream transcription factors that can target the *CUX1* gene. A bioinformatics analysis revealed a binding region for two transcription factors, *SP1* and *KROX20*, in the core promoter region of the *CUX1* gene. In addition, it has been reported that these two transcription factors play a regulatory role in hair follicle growth and development, and we speculate that the two transcription factors, *SP1* and *KROX20*, can target the *CUX1* gene [26,27,28].

Transcription factors are a class of DNA-binding proteins widely found in organisms that bind to specific DNA sequences. Many transcription factors have been reported to play crucial roles in hair morphogenesis. Raveh et al. found that inhibiting the expression of the transcription factor *Runx1* in the epidermis significantly deformed the structure of serrated hair in mice [38]. Additionally, the knockout of transcription factor *Runx3* and zinc finger transcription factor *Miz1* in mice resulted in a reduction in the amount of serrated hair in mice [39,40]. In this study, binding sites of transcription factors *SP1* and *KROX20* are known to exist in the *CUX1* core promoter region. Other studies have also demonstrated the significant involvement of transcription factors *SP1* and *KROX20* in regulating hair follicles. Transcription factor *Sp1* is crucial for the expression of many epidermal keratin intermediate filament genes, and transcription factor *KROX20* can regulate *Igfbp5* and thus affect hair morphology [26,27,28]. Consequently, we speculated that *SP1* and *KROX20* could regulate the transcription of the *CUX1* gene. We first found that *SP1* and *KROX20* could affect the transcription activity and mRNA expression level of *CUX1*, which indicated that *SP1* and *KROX20* may be two upstream transcription regulators for the *CUX1* gene. Additionally, we also found the positive influence of *SP1* and the negative influence of *KROX20* on the proliferation of DPCs, which is consistent with the regulatory role of the *CUX1* gene in DPC proliferation based on the regulatory effects of *SP1* and *KROX20* on the *CUX1* gene [10]. These findings further suggest that the role of the *CUX1* gene in DPC proliferation is regulated by the transcription factors *SP1* and *KROX20*.

## 5. Conclusions

In conclusion, this study preliminarily revealed the transcriptional regulation of the CUX1 gene by transcription factors Sp1 and KROX20, as well as the proliferation of DPCs in vitro regulated by these two transcription factors. These findings lay a theoretical foundation for further exploration of the mechanisms underlying CUX1 gene expression and proliferation regulation in DPCs.

## Figures and Tables

**Table 1:** Primer information used for qRT-PCR.

Gene	Primer Sequence (5′-3′)	Product Size (bp)	Annealing Temperature (°C)	Accession Number
*SP1*	F: GCAAGACCTCACACCTACGG R: GACACTCAGGGCAGGCAAAT	162	60	XM_027967251.2
*KROX20*	F: CACGTCGGTGACCATTTTCC	151	60	XM_027962248.2
	R: TGAGACCGGAGCAAAGCTG			
*CUX1*	F: GCACGACATTGAGACGGAG	160	60	XM_012104502.5
	R: AGCTATGGTCTCAGCCTGGT			
*PCNA*	F: CGAGGGCTTCGACACTTAC	97	60	XM_004014340.5
	R: GTCTTCATTGCCAGCACATT			
*CDK2*	F: AGAAGTGGCTGCATCACAAG	92	60	NM_001142509.1
	R: TCTCAGAATCTCCAGGGAATAG			
*cyclinD1*	F: CCGAGGAGAACAAGCAGATC	91	60	XM_027959928.2
	R: GAGGGTGGGTTGGAAATG			
*GAPDH*	F: TCTCAAGGGCATTCTAGGCTAC	151	60	NM_001190390.1
	R: GCCGAATTCATTGTCGTACCAG			

**Table 2:** The information of primers for constructing the SP1 and KROX20 overexpression vectors.

Primer Name	Primer Sequence (5′-3′)	Product Size (bp)	Annealing Temperature (°C)
pcDNA3.1-SP1	F: CTAGCGTTTAAACTTAAGCTTATGAGCGACCAAGATCACTCCATG	2361	65
	R: TGCTGGATATCTGCAGAATTCTCAGAAGCCATTGCCACTGATATTG		
pcDNA3.1-KROX20	F: CTAGCGTTTAAACTTAAGCTTATGCGAGTCGGGCTCCCCT	1458	65
	R: TGCTGGATATCTGCAGAATTCTCAGGGCGTCCGGGTCC		
pGL3-CUX1-1601	F: GCGTGCTAGCCCGGGCTCGAGTCCCCTGGACCAGCCTAGG R: CAGTACCGGAATGCCAAGCTTTCTTCCTCACTCCTAGTTGGTTCTG	1601	63

## Data Availability

All the data of this study are presented in the manuscript.

## References

[1] Houschyar K.S., Borrelli M.R., Tapking C., Popp D., Puladi B., Ooms M., Chelliah M.P., Rein S., Pforringer D., Thor D. (2020). Molecular Mechanisms of Hair Growth and Regeneration: Current Understanding and Novel Paradigms. Dermatology.

[2] Mok K.-W., Saxena N., Heitman N., Grisanti L., Srivastava D., Muraro M.J., Jacob T., Sennett R., Wang Z., Su Y. (2019). Dermal Condensate Niche Fate Specification Occurs Prior to Formation and Is Placode Progenitor Dependent. Dev. Cell.

[3] Jahoda C.A.B., Christiano A.M. (2011). Niche Crosstalk: Intercellular Signals at the Hair Follicle. Cell.

[4] Yang C.-C., Cotsarelis G. (2010). Review of hair follicle dermal cells. J. Dermatol. Sci..

[5] Nissimov J.N., Das Chaudhuri A.B. (2014). Hair curvature: A natural dialectic and review. Biol. Rev..

[6] Millar S.E. (2002). Molecular mechanisms regulating hair follicle development. J. Investig. Dermatol..

[7] Schneider M.R., Schmidt-Ullrich R., Paus R. (2009). The hair follicle as a dynamic miniorgan. Curr. Biol..

[8] Paus R., Epstein F.H., Cotsarelis G. (1999). The Biology of Hair Follicles. New Engl. J. Med..

[9] Driskell R.R., Clavel C., Rendl M., Watt F.M. (2011). Hair follicle dermal papilla cells at a glance. J. Cell Sci..

[10] Zhou H., Huang S., Lv X., Wang S., Cao X., Yuan Z., Getachew T., Mwacharo J.M., Haile A., Quan K. (2023). Effect of CUX1 on the Proliferation of Hu Sheep Dermal Papilla Cells and on the Wnt/beta-Catenin Signaling Pathway. Genes.

[11] Ellis T., Gambardella L., Horcher M., Tschanz S., Capol J., Bertram P., Jochum W., Barrandon Y., Busslinger M. (2001). The transcriptional repressor CDP (Cutl1) is essential for epithelial cell differentiation of the lung and the hair follicle. Genes Dev..

[12] Sinclair A.M., Lee J.A., Goldstein A., Xing D., Liu S., Ju R., Tucker P.W., Neufeld E.J., Scheuermann R.H. (2001). Lymphoid apoptosis and myeloid hyperplasia in CCAAT displacement protein mutant mice. Blood.

[13] Luong M.X., van der Meijden C.M., Xing D., Hesselton R., Monuki E.S., Jones S.N., Lian J.B., Stein J.L., Stein G.S., Neufeld E.J. (2023). Genetic Ablation of the CDP/Cux Protein C Terminus Results in Hair Cycle Defects and Reduced Male Fertility. Mol. Cell. Biol..

[14] Sansregret L., Goulet B., Harada R., Wilson B., Leduy L., Bertoglio J., Nepveu A. (2023). The p110 Isoform of the CDP/Cux Transcription Factor Accelerates Entry into S Phase. Mol. Cell. Biol..

[15] Truscott M., Denault J.-B., Goulet B., Leduy L., Salvesen G.S., Nepveu A. (2007). Carboxyl-terminal Proteolytic Processing of CUX1 by a Caspase Enables Transcriptional Activation in Proliferating Cells. J. Biol. Chem..

[16] Jing J., Xu P., Xu J.-L., Ding Y.-X., Yang X.-S., Jin X.-Q., Zhou L.-J., Chen Y.-H., Wu X.-J., Lu Z.-F. (2021). Expression and localization of Sox10 during hair follicle morphogenesis and induced hair cycle. Int. J. Med. Sci..

[17] Krieger K., Millar S.E., Mikuda N., Krahn I., Kloepper J.E., Bertolini M., Scheidereit C., Paus R., Schmidt-Ullrich R. (2018). NF-κB Participates in Mouse Hair Cycle Control and Plays Distinct Roles in the Various Pelage Hair Follicle Types. J. Investig. Dermatol..

[18] Tomann P., Paus R., Millar S.E., Scheidereit C., Schmidt-Ullrich R. (2016). LHX2 is a direct NF-κB target gene that promotes primary hair follicle placode down-growth. Development.

[19] Kadaja M., Keyes B.E., Lin M., Pasolli H.A., Genander M., Polak L., Stokes N., Zheng D., Fuchs E. (2014). SOX9: A stem cell transcriptional regulator of secreted niche signaling factors. Genes. Dev..

[20] Folgueras A.R., Guo X., Pasolli H.A., Stokes N., Polak L., Zheng D., Fuchs E. (2013). Architectural Niche Organization by LHX2 Is Linked to Hair Follicle Stem Cell Function. Cell Stem Cell.

[21] Kang X., Liu Y., Zhang J., Xu Q., Liu C., Fang M. (2017). Characteristics and Expression Profile ofKRT71Screened by Suppression Subtractive Hybridization cDNA Library in Curly Fleece Chinese Tan Sheep. DNA Cell Biol..

[22] Wang S., Wu T., Sun J., Li Y., Yuan Z., Sun W. (2021). Single-Cell Transcriptomics Reveals the Molecular Anatomy of Sheep Hair Follicle Heterogeneity and Wool Curvature. Front. Cell Dev. Biol..

[23] Livak K.J., Schmittgen T.D. (2001). Analysis of relative gene expression data using real-time quantitative PCR and the 2(-Delta Delta C(T)) Method. Methods.

[24] Jahoda C.A.B., Reynolds A.J., Chaponnier C., Forester J.C., Gabbiani G. (1991). Smooth muscle α-actin is a marker for hair follicle dermis in vivo and in vitro. J. Cell Sci..

[25] Wang S., Hu T., He M., Gu Y., Cao X., Yuan Z., Lv X., Getachew T., Quan K., Sun W. (2023). Defining ovine dermal papilla cell markers and identifying key signaling pathways regulating its intrinsic properties. Front. Vet. Sci..

[26] Dunn S.M., Keough R.A., Rogers G.E., Powell B.C. (1998). Regulation of a hair follicle keratin intermediate filament gene promoter. J. Cell Sci..

[27] Schlake T. (2006). Krox20, a novel candidate for the regulatory hierarchy that controls hair shaft bending. Mech. Dev..

[28] Duverger O., Morasso M.I. (2009). Epidermal patterning and induction of different hair types during mouse embryonic development. Birth Defects Res. Part C Embryo Today Rev..

[29] Lv X., Li Y., Chen W., Wang S., Cao X., Yuan Z., Getachew T., Mwacharo J., Haile A., Li Y. (2023). Association between DNA Methylation in the Core Promoter Region of the CUT-like Homeobox 1 (CUX1) Gene and Lambskin Pattern in Hu Sheep. Genes.

[30] Stenn K.S., Cotsarelis G. (2005). Bioengineering the hair follicle: Fringe benefits of stem cell technology. Curr. Opin. Biotechnol..

[31] Watabe R., Yamaguchi T., Kabashima-Kubo R., Yoshioka M., Nishio D., Nakamura M. (2014). Leptin controls hair follicle cycling. Exp. Dermatol..

[32] Avigad Laron E., Aamar E., Enshell-Seijffers D. (2018). The Mesenchymal Niche of the Hair Follicle Induces Regeneration by Releasing Primed Progenitors from Inhibitory Effects of Quiescent Stem Cells. Cell Rep..

[33] Yu D.W., Yang T., Sonoda T., Gaffney K., Jensen P.J., Dooley T., Ledbetter S., Freedberg I.M., Lavker R., Sun T.T. (1995). Message of nexin 1, a serine protease inhibitor, is accumulated in the follicular papilla during anagen of the hair cycle. J. Cell Sci..

[34] Driskell R.R., Giangreco A., Jensen K.B., Mulder K.W., Watt F.M. (2009). Sox2-positive dermal papilla cells specify hair follicle type in mammalian epidermis. Development.

[35] Chi W., Wu E., Morgan B.A. (2013). Dermal papilla cell number specifies hair size, shape and cycling and its reduction causes follicular decline. Development.

[36] Pierard-Franchimont C., Paquet P., Quatresooz P., Pierard G.E. (2011). Mechanobiology and cell tensegrity: The root of ethnic hair curling?. J. Cosmet. Dermatol..

[37] Lv X., Sun W., Zou S., Chen L., Mwacharo J.M., Wang J. (2019). Characteristics of the BMP7 Promoter in Hu Sheep. Animals.

[38] Raveh E., Cohen S., Levanon D., Negreanu V., Groner Y., Gat U. (2006). Dynamic expression of Runx1 in skin affects hair structure. Mech. Dev..

[39] Raveh E., Cohen S., Levanon D., Groner Y., Gat U. (2005). Runx3 is involved in hair shape determination. Dev. Dyn..

[40] Gebhardt A., Kosan C., Herkert B., Möröy T., Lutz W., Eilers M., Elsässer H.-P. (2007). Miz1 is required for hair follicle structure and hair morphogenesis. J. Cell Sci..

